# Cattle Reproductive Disorders Documented from Gaushalas of Nepal

**DOI:** 10.1155/2024/3058386

**Published:** 2024-04-23

**Authors:** Meena Pun, Bhuwan Raj Bhatt, Shambhu Shah, Narayan Neupane, Krishna Kaphle

**Affiliations:** ^1^Institute of Agriculture and Animal Science, Tribhuvan University, Kirtipur, Kathmandu, Nepal; ^2^Faculty of Agriculture, School of Agriculture, Far Western University, Tikapur, Nepal; ^3^Paklihawa Campus, Institute of Agriculture and Animal Science, Tribhuvan University, Bhairahawa, Nepal; ^4^Institute of Agriculture and Animal Science, Tribhuvan University, Kirtipur, Kathmandu, Nepal

## Abstract

Cow is the national animal of Nepal, yet it is one of the most abused animal species here. Under realized utilities of cow that is nonlactating or pregnant is the reason for demonic cruelty. Since the Vedic period, gaushalas have been caring for cows. At present, most gaushalas have responsibility to rescue, offer refuge, and treat poorly treated or confiscated cattle from smuggling rackets in Nepal. It is no surprise that these abused animals suffer from many health issues and compromised reproductive ability. This study was conducted to know about husbandry practice and to determine prevalence of reproductive disorders in cows of Gaushala from Nepal. Altogether, 27 gaushalas were visited throughout the study period and cows (≥2 years) (*n* = 2959) were included for the study. From the study, respondents from 14.81% Gaushala admitted indigenous cattle only, 11.11% admitted any breed (indigenous and crossbreed), 44.44% admitted stray animals only, and 29.63% admitted all types (indigenous, crossbreed, and stray animals). The study revealed that among (*n* = 2959) animals examined, 5.54% (*n* = 164) were affected by either one or more reproductive problems. The major reproductive disorders identified in study area were repeat breeding 0.47%, cervico-vaginal and uterine prolapse 0.34%, retention of placenta 2.13%, dystocia 0.61%, and abortion 1.66%. Herd size of Gaushala had a significant difference (*P* < 0.05) on the overall prevalence of reproductive problems in cows of Gaushala. The main issues with gaushalas included a lack of resources like adequate fund, feeds, fodder, and water, shortage of grazing acreage, veterinary services, and difficulties in managing male cattle. To mitigate the issues and welfare related to gaushalas strict adherence to disease surveillance and biosecurity rules, avoidance of unlimited reproduction in cows, and separation of males and females, fund raising and resource management, collaboration with local government and NGOs, veterinary hospital, clinics, research, and innovation with veterinary institution and universities.

## 1. Introduction

A “Gaushala” means a “home for cows,” especially housing bovines only, whereas “Pinjrapole” refers to the housing of all animals [1]. Gaushala is an institution established for the purpose of keeping, breeding, rearing, and maintaining cattle for the purpose of reception, protection, and treatment of infirm, aged, or diseased cattle. It is primarily focused on providing shelter to cows and caters mostly to the needs of nonlactating, weak, unproductive, and stray cattle [2].

Cow shelters or cow sanctuaries called “Gaushalas” or “Gau Sadans” are the place where abandoned and unproductive, and old cows are housed by philanthropists, animal protection organizations, religious organizations, and temple trusts [3]. These cow shelters are traditional and ancient rescue homes for cows with documentary evidence of their existence since the 3^rd^ to 4^th^ century B.C. [4]. The sacredness and high ritual status of the cow have led to the use of “panchgavyas” or the five cow products: milk, curd, butter, urine, and dung, for the maintenance of a person as free from pollution and for the purification rituals in Hindu religious ceremonies [5].

Cow is the national animal of Nepal, and its abuse and exploitation are not in harmony with societal anticipation. Cow being a multiutility animal in every society is more equal than other animals' status in Nepal and even the dung and urine are used in rituals and medicinal purposes. Welfare and rights of farm animals: Several approaches are being discussed such as the Western norm of welfare and rights (freedom) of farm animals and the Sanatani ways of all animal well-being, with some species being worshipped and demanded that they be taken care of.

In Nepal, indigenous cattle are small sized and humped (*Bos indicus*) with the exception of Lulu cattle, which is hump less. They are disease-resistant and hardy in nature and survive in a poor pasture and harsh weather. They can survive in scarce condition without supplement of extra concentrate ration. Sanatan Omkar family is the religion of almost all of Nepal (over 85%), and cow is a divine being. Abode of all Gods and Goddesses, cow is in the form of Laxmi and Bull the ride of the nation protecting God—Lord Pashupatinath.

Nepal's policy to patronage high-yielding exotic breeds of dairy cattle has proven to be miscalculated move. Management of nonproductive animals was not carefully considered, and the farmers and community are paying for it. Revival of interest in indigenous cattle breed selection and conservation in Nepal is interesting fact. Although their milk production is lower than other exotic cattle breeds, they are very useful in many aspects. The total cattle population in Nepal was 73,85,035 in Nepal (MoLD, 2019). There were about 1,200 road cattle in Kathmandu valley [6], and they pose serious health and safety risk [7]. Lobago et al. [8] described that among the major reproductive problems that have direct impact on reproductive performance of dairy cows are abortion, dystocia, RFM, pyometra, metritis, prolapse (uterine and vaginal), anestrus, and repeat breeding.

Old cows after cessation of productive life have several fates. Various types of reproductive problems have been reported in the dairy cows. Even after treatment, if the cow does not conceive, the farmers tend to dispose the cow to the traders, let them go free roaming in the roadside or left in the nearby jungle. This has been causing some sorts of social conflicts and threat to the wildlife and conservation of forest as well. In the city, leaving such unproductive cattle free in the road side has also cause problems in the traffic management in the major cities. In Nepal, there is prohibition of cattle slaughter in the country's law and there is people sentiment too, and the disposal of such unproductive animals has been a real problem to the dairy animal farmers and creates economic burden to farmer for maintaining them in the herd [9].

Majorly, unproductive, old, and stray cattle find shelter in the gaushalas instead of individual households. This tragic plight of the stray cows is a consequence of uneconomical returns due to low productivity and replacement of draft power in agriculture by mechanization. The rural people own cows despite having limited land to graze them but nowadays due to urbanization has encroached upon the traditional grazing lands leading to cows roaming freely in the streets, raiding crops, suffering automobile hits, and causing traffic problems. In the cities, these street cows survive on roadside city garbage that is contaminated with plastics that leads to health issues causing painful deaths. There have been reports of many fatal road accidents due to automobile accidents involving cattle in the streets [10].

Proper Gaushala management is very important for its long-term performance. It has been found that initiation has been taken to mitigate stray cattle problem by establishing gaushalas. These are the protective shelters for stray, abandoned, handicapped, and infirm cattle. Management of cows in Gaushala can prevent road accidents and crop damage, and prevent premature death of these cattle due to consumption of polythene bags along with that they also provide rescue and treatments of sick, injured, and accidental animals. The challenge to keep the welfare of these animals at its peak, normal behavior expression, and access to religious/community forests and also timely vaccinate and provide access to health care is in practice, but disease outbreaks do happen [11].

In the recent article by [12], meat adulteration was found by slaughtering stray cattle as supply of buffaloes was not possible in this pandemic condition. This problem is serious concern for animal welfare too and ticking time bomb for social unrest. Despite of huge importance of Gaushala, not many systematic studies were conducted to find out problem of reproductive disorder in cattle and general management practice in gaushalas of Nepal. Therefore, this survey study will collect and analyze information about animal husbandry practices and operations, which help to understand sociodemographic characteristic, husbandry/management practices, reproductive disorders in response to feeding, and herd health of gaushalas of Nepal.

## 2. Materials and Methods

### 2.1. Study Area

**Figure 1:**
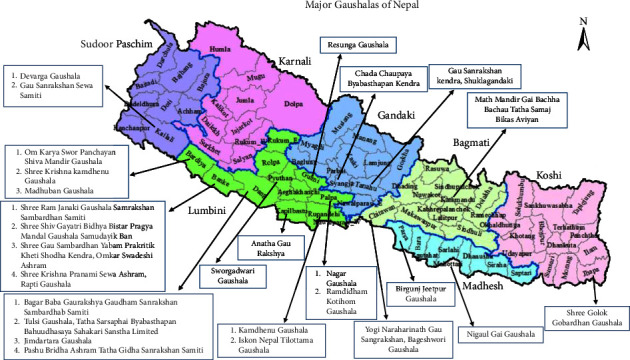
Study site showing major Gaushalas of Nepal.

Nepal is of roughly trapezoidal shape, about 800 km long and 200 km wide, with an area of 1,47,516 km^2^ (see Figure 1). It lies between latitudes 26° and 31° North, and longitudes 80° and 89° East. Nepal has a diverse geography, including fertile plains and subalpine forested hills.

### 2.2. Sample Size, Sampling Population, and Sampling Procedure

There is no record of Gaushala number present in Nepal. So, snow ball sampling technique was used as sampling technique. Altogether, 27 Gaushalas were visited throughout the study period and cows (≥2 years) (*n* = 2959) were included for the study.

(i) *Sample size*: As the study was based on snow ball sampling technique, the first respondent was the Gaushala registered with the Social Welfare Council (SWC) as well as those that were reachable during the study period.
  - Total no. of Gaushala (*n* = 27)
  - Total no. of cows (≥2 years) (*n* = 2959)
Information on various aspects of Gaushala was collected by face-to-face interview. The information and data mainly contain:
(ii) *Sociodemographic parameter*
  1. Gender
  2. Age
  3. Education level
  4. Ethnicity
  5. Religion
  6. Job role at Gaushala
  7. Duration of involvement at Gaushala
(iii) *Husbandry practice parameter*
  1. Types of animals admitted in Gaushala
  2. Number of animals in Gaushala
  3. Production of gaumutra (arka)
  4. Deworming status
  5. Vaccination status
  6. Provision of extra concentrate to pregnant and lactating cattle
  7. Provision of mineral mixture powder
  8. Cultivation of green fodder
  9. Sale of milk
(iv) Reproductive disorder in cattle parameter
Number of affected cattle from various reproductive disorders (retention of placenta, abortion, dystocia, repeat breeding, vaginal prolapse, uterine prolapse).

### 2.3. Data Collection

Primary data were collected from the interview of Gaushala manager through face-to-face interview. It consists of multiple choice, semiclosed, closed, and open-ended questions. Secondary data were collected from different articles, journals, and reports of different organizations like MoALD, SWC, and other institutions of the study area. Data collection was done in winter season from November 2020 to February 2021.

### 2.4. Methods and Techniques of Data Analysis

Microsoft Excel 2016 was used for data entry. Both descriptive statistics and inferential statistics were used for data analysis. The data collected were coded tabulated, analyzed, and interpreted using descriptive tools like frequency, percentage, and mean. Tables, figures, and graphs were used for the presentation of data. *P* value and chi-square value were calculated by Open EPi software. Significant difference was declared for means with *P* < 0.05.

## 3. Results and Discussion

### 3.1. Sociodemographic Characteristics

Among the total respondents, 85.19% were males and 14.81% were females. Age of respondents was 18–25 (7.41%), 26–35 (14.81%), 36–45 (29.63%), 46–55 (33.33%), and 56–65 (14.81%). Of the respondents, the distribution of education level was as follows: 7.41% had no formal education, 44.44% had education below the 10th class, 22.22% completed the 10th class, 14.81% had completed the 10+2 class, and 11.11% were graduates. Major respondents were Brahmin 44.44%, Chhetri 33.33%, Janajati 18.52%, and Dalit 3.70%. All of the respondents followed Hinduism. According to the study, information obtained was 37.04% respondents directly worked with animal and 62.96% worked as team leader. The respondents tenure at Gaushala was ≤3 years (48.5%), 3–5 years (29.63%), 5–9 years 11.11%), 10–15 years (3.70%), and more than 15 years (7.41%) as shown in Table 1.

### 3.2. Husbandry Practice

#### 3.2.1. Types of Animals Admitted in Gaushalas

This study shows 14.81% Gaushala admitted indigenous cattle only, 11.11% admitted any breed (indigenous and crossbreed only), 44.44% admitted stray animals only, and 29.63% admitted all types (indigenous, crossbreed, and stray animals) (see Table 2).

#### 3.2.2. Number of Animals in Gaushala

Among all 4521 numbers of animals in Gaushalas, highest number of animals 2959 were above 2 years of age followed by 567 numbers were bull, below 6 months of female and male calves were 387 and 355 and 253 were heifers below 2 years of age (see Table 3).

#### 3.2.3. Status of Gaumutra (Arka) Production, Deworming, Vaccination, Extra Ration Feeding during Pregnancy, Mineral Mixture, Green Fodder Cultivation, and Sale of Milk

Gaumutra (arka) was produced by 33.33% and 66.67% of Gaushala did not produce gaumutra. Deworming practice was followed by 40.07%, and 59.26% did not practice deworming of animals. Vaccination practice against different diseases like FMD, HS, and BQ was in 37.04%, and 62.96% Gaushala did not vaccinate animals. Feeding of extra ration during pregnancy was practiced by 59.26% and did not practice by 40.74% of Gaushala. Provision of mineral mixture powder was practiced in 18.52% and did not practice in 81.48% of Gaushala. Green fodders like oat and napier were cultivated in 44.44% and did not practice green fodder cultivation in 55.56% of Gaushala. Sale of milk was practiced in 14.81% Gaushala and did not practice in 85.19% of Gaushala. Total lactating animals in Gaushala were 186, and total milk production (ltrs/day) was 267.5 (see Table 4).

### 3.3. Overall Prevalence of Reproductive Disorders in Cows of Gaushalas' Total Sample (*n* = 2959)

In the present study, 5.54% (*n* = 164) (see Table 5) of cattle in the study areas were affected by either one or more reproductive disorders, which is similar to 6.06% reported by Singh et al. [13] but lower than 33.85% reported by [14] in dairy cattle of India, 33.45% reported by [15], 11.7% reported by [16], and 21.40% reported by [17]. This difference in prevalence in reproductive disorders might be due to variations in predisposing factors, including nutritional status and management [18], and also may be due to variations in sample size, production system, study methodology, and breed of animals, as well as environmental factors [19].

### 3.4. Prevalence of Reproductive Disorders in Cattle of Gaushalas

The major reproductive disorders identified in study area were repeat breeding 0.47%, cervico-vaginal prolapse 0.34%, uterine prolapse 0.34%, retention of placenta 2.13%, dystocia 0.61%, and abortion 1.66% (see Table 6).

#### 3.4.1. Retention of Placenta

The higher prevalence of retention of placenta, 2.13%, was found in this study, which is similar to 3.8% reported by Hadush et al. [20]; 2.1% reported by [21] in dairy cattle of Ethiopia and 1.65% reported by [22], and lower than the 13.4% reported by [23] in dairy cows of Bangladesh, 13.75% reported by [15]. The variation in the prevalence of ROP might be attributed due to the presence of infection, dystocia and its predisposing factors, disease conditions, and management difference, especially feeding and sanitation [24].

#### 3.4.2. Abortion

The prevalence rate of abortion 1.66% recorded in this study is similar to the 2.23%, 2.56%, and 2.9% reported by Bekana et al. [25, 19] and Tulu and Gebeyehu [16] in Ethiopia, respectively, and lower than 5.68% reported by [26] in dairy cattle, India; 6.32% reported by [15] in chauries of Nepal; and 8.16% reported by [27] in cattle of Bangladesh. The differences in abortion prevalence can be attributed by various factors such as breed, management systems (especially feeding and standard practices), overcrowding, and intra-group aggression leading to traumatic abortions [28].

#### 3.4.3. Dystocia

The prevalence rate of dystocia 0.61% observed in this study is in line with 0.32% reported by [13] and 1.26% reported by [29] and lower than 2.19% reported by [26], and 5.7% by [30]. This variation in the prevalence of dystocia is influenced by various factors, such as the age and parity of the dam as well as breed of the sire [31], size of bull used, and fetus and birth canal of dairy cattle in different study areas [30].

#### 3.4.4. Repeat Breeding

The prevalence of repeat breeding 0.47% in the present study is similar with the 0.71% reported by [32], and 0.5% reported by [21]. But lower than 7.27% reported by [33] in cow of Pakistan, 4.39% reported by [17] in cattle of India, and 5.20% reported by [15]. This variation might be due to number of factors, including subfertile bulls, endocrine imbalance, malnutrition, reproductive tract infections, and poor management practices, such as faulty heat detection and communal use of bull for natural services [34].

#### 3.4.5. Vaginal Prolapse

The prevalence rate of vaginal prolapse 0.34% recorded in this study is similar to 0.7% reported by [21] but is lower than the 1.24%, 1.95%, and 2.05% reported by [19, 20, 35], respectively. This variation might be due to management system (feeding), sample size, and breed of animals [19].

#### 3.4.6. Uterine Prolapse

The prevalence of uterine prolapse 0.34% observed in this study is similar with 0.76% reported by [19] but lower than 1.6% reported by [36] and 2.1% reported by [37]. This variation might be due to environmental and management factors [37]. Forced extraction of the fetus is incriminated as an etiological factor for uterine prolapse [38].

### 3.5. Prevalence of Reproductive Disorders in Cattle at Different Provinces of Nepal Total sample (*n* = 2959)

**Figure 2:**
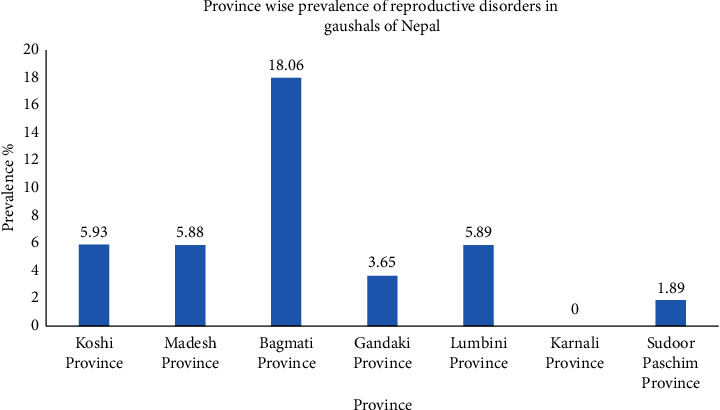
Prevalence of reproductive disorders in cattle from different provinces of Nepal.

According to the study, higher prevalence of reproductive disorders in cattle of Gaushala was observed in Bagmati Province (18.06%), Koshi (5.93%), Lumbini (5.89%), Madhesh (5.88%), Gandaki (3.65%), and Sudoor Paschim Province (1.89%) (see Figure 2). This variation in prevalence of reproductive problems might be due to difference in sample size and management factors, and the presence of stray animals affected from different reproductive problems.

### 3.6. Prevalence of Reproductive Disorders Associated with Herd Size

Herd size risk factor was associated with the occurrence of reproductive problems in cows. From the study, among total animals examined, *n* = 191 cows were from small herd size (≤50), *n* = 759 cows were from medium (51 to 150) herd size, and *n* = 2009 were from large (>150) herd size (see Table 7). According to the study, among the cows examined, reproductive disorder in small herd size was 7.85%, in medium herd size 7.38%, and in large herd size 4.63% (see Table 7). Herd size of Gaushala had a significant difference (*P* < 0.05) on the overall prevalence of reproductive problems in cows of Gaushala. Small herd size was having significantly higher reproductive problems than others.

The result of the current study is different than prevalence of reproductive disorders, 12.6% in large herd size, and 11.1% in small herd size reported by [30]. This variation might be due to differences in environmental factors, sample size, breed of cattle, and level of veterinary services [30], variations in management practices, and hygienic condition, which differ from time to time and place to place [29].

### 3.7. Prevalence of Reproductive Disorders Associated with Feeding Practice

From the study, among total animals examined, *n* = 311 cows were stall fed, *n* = 1442 cows were grazed, and *n* = 1206 practiced both feeding systems (see Table 8). According to the study, among the cows examined, reproductive disorders in stall feeding were 7.40%, grazing 4.79%, and both feeding practices 5.97% (see Table 8). There is no significant difference (*P* > 0.05) of feeding practice on the overall prevalence of reproductive problems in cows of Gaushala. Stall feeding practice cows were having higher reproductive problems than others.

Similarly, incidence of reproductive disorders was more frequent in intensively managed farms as compared to semi-intensively managed one [32] but different from higher prevalence of infertility problems also observed in animal using grazing methods of feeding practice 26 (59.1%) than stall and both types of feeding practice [24]. This variation might be due to crowdedness and the poor hygienic conditions of intensively managed farms [32]. The expression of natural behaviors, such as eating and resting, is suppressed [39], and various production diseases, such as lameness, mastitis, and hock lesions, occur in stall housing systems [40].

Stray cattle pose a nationwide challenge in Nepal's livestock industry. Abandoned due to low productivity, disease, disability, or old age, these animals often find themselves wandering in jungles and along roadsides. While gaushalas in Nepal do play a crucial role in managing some of these stray cattle, their capacity falls short of accommodating all abandoned animals. Despite efforts, husbandry practices within gaushalas lag behind modern scientific standards due to limitations in economic resources and skilled manpower.

The government at various levels—central, provincial, and local—is involved in addressing the issue of stray cattle. It is imperative to ensure animal welfare and uphold the five freedoms of animals, without imposing an undue burden on society. Stray animals can be utilized economically in various ways: (a) biogas production, (b) use of dung as organic manure, (c) utilization of urine for fertilizers, (d) distillation of urine for medicinal purposes, (e) ghee extraction for medicinal use, (f) production of cow dung agarbatti (incense sticks), (g) utilization of dried cow dung as fuel, (h) production of panchagavya, a traditional concoction, and (i) promotion of tourism around indigenous cattle conservation sites for both local and foreign visitors. Efforts should be made to integrate these approaches effectively to address the issue of stray cattle while promoting sustainable practices and economic viability.

## 4. Conclusion

The study explored 5.54% prevalence of reproductive disorders in cows. Results of this study revealed that highest prevalence of retention of placenta followed by abortion, dystocia, repeat breeding, cervico-vaginal, and uterine prolapse, respectively. Lack of funds, unavailability of sufficient feeds and fodder, lack of grazing land, accidents while grazing at hilly areas, lack of scientific housing system, lack of water in some gaushalas and some gaushalas were also facing registration problems, lack of veterinary services, difficulty in male cattle management, etc., were the major problems in gaushalas. Only prevalence of reproductive disorders was investigated in this study. More emphasis should be given for proper management of gaushalas. Gausevak's (cow herders) retention was noted as one of the most reported hurdles at the gaushalas. Nominal payment, the wandering interest of the hermits, lack of proper feeding, accommodation, and healthcare facilities are diagnosed as the major cause of disinterest.

Pensions for retired animals, retirement houses, ecological services, etc., are some approaches suggested here in Nepal. The government and farming community are insensitive as their greed for-profit and insensitiveness for the issue are continuing abuse of the animals and weak regulation is allowing animal abandonment, but with vigilant community members on patrol and adoption of individual animal tracking tools in hand, several farmers were fined heavily for their cruel act of abandoning old and unproductive animals. Slaughter is being advocated by certain sections of media, some communities, and religious tourism lobby groups, but it is not a wise suggestion from Nepal because (a) Nepal's majority are nonbeef consumers; in contrary, they worship cows on daily or ceremonial occasions; (b) Retransformative religious tourism is the main prospect for Nepal's sustainable transformation and this penny to be benefited from cow slaughter will hurt the pound to be earned from visits by religious tourism that find every river and hills of Nepal to be holy and every rock and water to be divine; (c) the assumption that the male and unproductive exotic breed of dairy animals will find the market in Bangladesh and elsewhere is wrong and flawed; and (d) the free and open border down south (Indian border) and mass migration from there is stretching resources for management here in Nepal, but with time and arrangements in India, our small efforts can take care of Nepal's need for housing and care. Thus, we attempt to bring to notice the issue that is complex from so many dimensions, and as veterinarians, we focus on the management and reproductive issues of such managed cows. The sensitive issue needs to be brought to the front for wider discussion and as references for further reference.

## Figures and Tables

**Table 1:** Demography of respondents representing various gaushalas in Nepal includes variables, such as gender, age, education level, ethnicity, religion, job role, and duration of involvement at gaushalas.

Variables	No. of respondents (%)
*Gender*	
Male	23 (85.19)
Female	4 (14.81)

*Age*	
18–25	2 (7.41)
26–35	4 (14.81)
36–45	8 (29.63)
46–55	9 (33.33)
56–65	4 (14.81)

*Education level*	
No formal education	2 (7.41)
Below 10^th^ class	12 (44.44)
10^th^ class	6 (22.22)
10 + 2 class	4 (14.81)
Graduate	3 (11.11)

*Ethnicity*	
Brahmin	12 (44.44)
Chhetri	9 (33.33)
Janajati	5 (18.52)
Dalit	1 (3.70)

*Religion*	
Hinduism	27 (100)

*Job role at Gaushala*	
Work directly with animals	10 (37.04)
Team leader: supervise people who work directly with animals	17 (62.96)

*Duration of involvement at Gaushalas*	
3 years	13 (48.5)
3–5 years	8 (29.63)
5–9 years	3 (11.11)
10 to 15 years	1 (3.70)
More than 15 years	2 (7.41)

**Table 2:** Types of animals admitted in the Gaushalas.

Variables	No. of Gaushala (%)
Indigenous only	4 (14.81)
Any breed (indigenous and cross breed only)	3 (11.11)
Stray animals only	12 (44.44)
All (indigenous and stray animals)	8 (29.63)

**Table 3:** No. of animals housed in the Gaushala.

Variables	Total no.	Mean
Total no. of bulls in gaushala	567	21
Total no. of cows (≥2 years)	2959	109
Total no. of heifers (≤2 years)	253	9
Total no. of male calves (below 6 months) in gaushala	355	13
Total no. of female calves (below 6 months) in gaushala	387	14
Total no. of animals in Gaushala	4521	167

**Table 4:** Status of gaumutra (arka) production, deworming, vaccination, extra ration feeding during pregnancy, mineral mixture, green fodder cultivation, and sale of milk.

Variables	Production (%)	
	Yes	No
Gaumutra	33.33	66.67
Deworming	40.07	59.26
Vaccination	37.04	62.96
Feeding extra ration during pregnancy period	59.26	40.74
Provision of mineral mixture powder	18.52	81.48
Cultivation of green fodder practice	44.44	55.56
Sale of milk	14.81	85.19

**Table 5:** Overall prevalence of reproductive disorders in cows of gaushala.

Status of animals	No. of animal	Overall prevalence (%)
Animals with RDs	164	5.54
Animals without RDs	2795	94.46
Total animal	2959	5.54

**Table 6:** Prevalence of reproductive disorders in cattle of Gaushala.

Types of reproductive disorders in cows	Number of affected cows	Prevalence (%)
Repeat breeding	14	0.47
Cervico-vaginal prolapse	10	0.34
Uterine prolapse	10	0.34
Retention of placenta	63	2.13
Dystocia	18	0.61
Abortion	49	1.66
Total	164	5.54

**Table 7:** Prevalence of reproductive disorders associated with herd size.

Variables	No. of cows	Affected no.	Nonaffected no.	*P* value	Chi-square value
Small (≤50)	191	15 (7.85%)	176 (92.15%)	0.01	8.96
Medium (51 to 150)	759	56 (7.38%)	727 (95.78)		
Large (>150)	2009	93 (4.63%)	1892 (94.18)		

**Table 8:** Prevalence of reproductive disorders associated with feeding practice.

Variables	No. of cows	Affected no.	No-affected no.	*P* value	Chi-square value
Stall	311	23 (7.40%)	288 (92.60%)	0.16	3.5
Grazing	1442	69 (4.79%)	1373 (95.21%)		
Both	1206	72 (5.97%)	1134 (94.03%)		

## Data Availability

The data used to support the findings of this study are available from the corresponding author upon request.
